# Theorizing Technological Mediation in the Outdoor Classroom

**DOI:** 10.1007/s42438-022-00315-2

**Published:** 2022-06-07

**Authors:** Imre van Kraalingen

**Affiliations:** Department of Teacher Education and Outdoor Studies, Norwegian School of Sport Sciences, Oslo, Norway

**Keywords:** Outdoor education, Postdigital, Mobile technology, Mlearning, Postphenomenology, Mediation theory

## Abstract

Situated within the context of the changing nature of teaching and learning in a postdigital context, this paper aims to theorize the mediating impacts of mobile technologies on outdoor learning experiences. Technological mediation is arguably a vital, yet often neglected, aspect of pedagogical practices. Today, the increasing employment of mobile technologies is not only changing the practices of outdoor education, but also challenging the traditional values of the field. This paper calls the predominant view that technology places a barrier between learners and the environment into question and offers a novel theoretical perspective. Inspired by postphenomenological mediation theory, the paper proposes a tri-polar technological mediation and outdoor learning framework. The framework offers a deeper understanding of the different dimensions of the mediating impacts of mobile devices on the relations between learners, their peers, and the natural environment in the outdoor classroom.

## Introduction

Current issues in the field of outdoor education can only be fully understood in the context of the fundamental societal and cultural transformations that are taking place in today’s societies. Thus, situated within the context of the changing nature of teaching and learning in a culture that is progressively described as ‘postdigital’ (Feenberg 2019; Jandrić et al. 2018, 2019; Reed 2021), this paper aims to gain a deeper understanding of how mobile technologies mediate outdoor learning experiences.

The term ‘postdigital’ indicates that we have moved beyond the novelty of digital technologies and recognizes that such technologies are ‘already embedded in, and entangled with, existing social practices and economic and political systems’ (Knox 2019: 358). As such, digital technologies are understood to no longer be ‘separate, virtual, “other” to a “natural” human and social life’ (Jandrić et al. 2018: 893; MacKenzie et al. 2021).

In line with these broader processes of change, the large-scale uptake of digital and mobile technologies as learning tools is rapidly expanding across educational practices and research (Pachler et al. 2009: 3). In addition to the learning activities and content, it is argued that education programs must consider the role and impact of technological developments on learning (Zidoun et al. 2019).

Against this backdrop, debates on how modern technologies are influencing the field, and how to manage the use of such technologies, have dominated the outdoor educational discourse (Greenwood and Hougham 2015; Hills and Thomas 2021; van Kraalingen 2021). However, the employment of mobile technologies in outdoor education has remained a debated and contested issue (Hills and Thomas 2021), as the use of such devices might conflict with the traditional objectives of outdoor education. In particular, concerns have been raised regarding how mobile technologies might influence human-nature and human–human interactions in the outdoors and stand in the way of a direct and sensory experience of the natural world (Greenwood and Hougham 2015; Smith et al. 2016). It is *not* the aim of this paper to provide guidelines for *how* mobile technologies should be used in the outdoor classroom, but rather to provide a deeper, more theoretical understanding of these mediating influences in a way that has not been done before. The novelty of the theoretical approach taken in this paper can be considered the principal contribution to existing knowledge of the use of educational technology in outdoor education.

The embeddedness of modern technologies in nearly every facet of people’s everyday life has led contemporary theory to predominantly describe technologically mediated experiences as ‘disembodied’ (Verhoef and Du Toit 2018). Interactions with and through mobile devices clearly carry a notion of a disembodied telepresence that is seemingly endemic to digital screen media, as we are frequently on-the-move, on-the-street, and purposefully situated in local spaces and places when engaged in mobile phone use (Richardson and Wilken 2012: 184). The meaning-making that transpires through embodied and sensory engagement does not merely happen between the body and the environment by means of how we are in and engage with a place, or how we perceive and organize the sensory input (Spinney 2007: 25–26), but also increasingly depends on ‘how they are mediated by other everyday entities and technologies’ including and, indeed, especially mobile technologies (Wilken and Goggin 2012: 12).

In this regard, I argue that the main pedagogical consideration is not to reach a moral conclusion on the rights or wrongs about the use of various mobile technologies, rather to acknowledge and more precisely understand their mediating role and impact on our experience of the world. Thus, it may be time for the field of outdoor education to move away from the ‘rights’ and ‘wrongs’ debates. van Kraalingen (2021: 12) calls for precisely this: for the field ‘to overcome and reconcile the nature-technology dichotomy.’

The contribution of this paper is to deepen these critiques and explore new theoretical underpinnings that can help better understand the complex nature of technological mediation of user engagement with mobile devices in the outdoor classroom. First, the role of mobile technologies in outdoor education will be addressed. Thereafter, I will discuss the limited capability of phenomenological tool analysis to fully explain the complexities of the mediating impact of modern technologies. Finally, I will arrive at exploring a postphenomenological perspective on technological mediation and suggest a new framework for understanding technological mediation in the context of outdoor education. More broadly, this theoretical inquiry aims to bridge the looming gap between philosophy and practice.

## Learning with Mobile Technology

Learning with mobile technologies, also known as mobile learning or ‘mlearning’, is rapidly expanding across educational practices and research (Pachler et al. 2009: 3). Crompton (2013: 4) defines mlearning as ‘learning across multiple contexts, through social and content interactions, using personal electronic devices’. Differently, Romrell et al. (2014) write that mlearning is learning which is situated, personalized, and connected through using mobile technologies. They further explain that mlearning is already taking place when someone accesses online networks from an iPad, for example, to answer a question. Mlearning would then also be taking place if a learner uses a smartphone to plan a hiking route using a digital map. Brown and Mbati (2015) point out that mlearning is not solely linked to mobile phones, but includes various other mobile technologies, including their software applications.

The elementary principles informing research on mobile learning are not novel. Pachler et al. (2009: 6) underscore that learning mediated by and through technology ‘is a hugely contested and much written about field’. However, what is new are the advancements in the (multi-)functionality of mobile technologies and the convergence of services afforded by a single device (Pachler et al. 2009). Indeed, mobile devices have arguably evolved into the first wireless, personal computers; they enable wireless and mobile computing capabilities combined with communication technologies (Jarvenpaa and Lang 2005). Different from other computers (such as desktop or laptop), the immediate site of mobile devices, whether held in the hand, close to the face, or connected via a headpiece, is generally close to the body (Jarvenpaa and Lang 2005). More than being an object ready-at-hand, the attachment to mobile technology reveals through being almost always in the hand (Malpas 2012: 30). This is a distinct feature of such devices, which can lead them to be understood as an extension of the self (Malpas 2012: 30), but also as an extended self (Belk 2013), which can be present in physical and virtual spaces at the same time (Belk 2013; Traxler 2010).

Mobile technologies of various kinds (i.e., smartphones, iPads, GoPros) have two obvious characteristics: mobility and portability. These features enable corporal mobility in terms of using the device almost anywhere and at any time, and also while being on the move, they allow devices to be connected nearly anytime and anywhere to the network (Casey 2012; Traxler 2010; Wilken and Goggin 2012). In this regard, Traxler (2010) argues that educational practices are no longer bound by conventional time and place boundaries. He further underlines that education systems need to change in order to align to the ways in which many students today perceive the world they live in and adapt to changes in an increasingly mobile society.

Mobile devices can be valuable learning tools for the outdoor classroom, given their mobility and multifunctionality. Such devices enable communication, access to, and the documentation of, information—words, images, videos—that can be shared and transferred anywhere or looked at later in time (Casey 2012: 179). That said, van Kraalingen (2021) also highlights various pitfalls, such as technologies becoming the focus point in learning activities, a decrease of learners’ skill development without the use of technology, and an interference with learners’ direct experience of nature. These pitfalls may lead to a mismatch between the use of mobile technologies and the learning objectives of outdoor education.

Outdoor educators have long advocated the benefits of outdoor education in terms of developing a connection to nature, personal and social development, simplicity, silence, cooperation, and so forth (see for example, Ewert 1989; McRae 1990; Wattchow 2001). These outdoor educational practices, theories, and philosophies are founded on ‘assumptions regarding the need for experiences that provide a balanced curriculum or purport to serve as an antidote to the ailments of modern life’ (Wattchow 2001: 20). With regard to the above, it can be interpreted that outdoor educators, practitioners, and researchers share the same concern as those involved in other educational contexts, as described by Pachler et al. (2009), namely, how learning is mediated by and through technology.

Nonetheless, the employment of mobile technologies as learning tools for pedagogical purposes is on the rise worldwide (Norris and Soloway 2015; Schilhab et al. 2018). This development has been reinforced during the Covid-19 pandemic (MacKenzie et al. 2021). Outdoor education has not remained immune to the processes of consumption, intensification, and commodification (Loynes 1998; Payne 2002; Payne and Wattchow 2008). Various authors have criticized the mismatch between the contemporary practice of outdoor education and its traditional philosophical underpinnings. According to Payne and Wattchow,

…a vicious cycle seems to be occurring where school-based outdoor education is a reflection of the faster cultural and technological phenomena. It becomes increasingly difficult to confidently make the claim that outdoor education is an ‘alternative’ beyond the fact that some of it occurs in the outdoors. (Payne and Wattchow 2008: 26)

From a broader, postdigital perspective, which considers the far-reaching consequences of rapid technological transformations in our contemporary world, Traxler et al. (2022: 497) argue that ‘developed in times and contexts very different from ours, traditional philosophies … need to be reimagined and repurposed for today’s challenges’. Consequently, the increasing use of mobile technologies in the outdoor classroom and the ways in which they mediate outdoor learning experiences, including human-nature and human–human interactions, are worthy topics of investigation.

## Phenomenology and Technology

Before turning to a discussion on phenomenology and technology, it is important to clarify that this paper does not intend to unravel the complex ontological principles of, and critiques on, phenomenology and postphenomenology. For the purposes of this paper, the discussion’s focus lies on the ability of these two philosophies to contribute to a more exacting capture of the mediating impact of modern, mobile technologies on human-world relations. Thus, this paper approaches phenomenology and postphenomenology as philosophies, rather than methodological paradigms.

There exists a widely held belief that outdoor learning is best nurtured through approaches that focus on direct experiences with nature (Braun and Dierkes 2017; Priest 1986; Wattchow 2004). Traditionally, phenomenology has been an appealing philosophy and methodology for researchers in the field of outdoor studies due to its potential to understand how people experience the world through their direct, first-hand experience (Allen-Collinson and Leledaki 2015; Nicol 2014; Telford 2019). Yet, in today’s postdigital context (Reed 2021), our engagement with digital and mobile technologies is increasingly shifting our direct experience of the environment to a human-technology-nature interface.

The argument is not that phenomenologists have neglected the presence and influence of technology. Although his perspective on technology was tinted rather negatively, Heidegger paid quite a lot of attention to the matter. Similarly, Merleau-Ponty has included accounts of concrete technologies in his discussion of embodiment. So, both Heidegger (1996) and Merleau-Ponty (1962) have—to various extents—discussed the ways technologies alter our being-in-the-world. In particular, Heidegger’s *tools analysis* can be considered an applicable analytical approach to understanding the role of technologies, as it has offered an important phenomenological understanding of how tools are used (Schmitt 1965). In *Being and Time*, Heidegger (1996) famously argued that ordinary tool use does not consist in interacting with material objects, but in skillfully dealing with things and putting them to use. In such skillful coping, tools and technologies are characterized by experiential transparency, in the sense that they withdraw from conscious awareness and allow us to focus on the task. Put differently, one may use a tool, without any particular awareness of its properties, but rather as if one ‘sees through’ them in order to perform the task one is engaged in. Heidegger utilizes the now-famous concept of ‘ready-to-hand’ to describe this specific mode of being (Rosenberger and Verbeek 2015: 15). I will not dwell on Heidegger’s ontological questioning of what tools are *in themselves* (more than their materiality) or whether they *are* independent from human intentionality. The point I am making is that phenomenological tool analysis is largely focused on what tools are and how they are used.

Phenomenology has been criticized for its romantic character (Ihde 1995), naïve empiricism (Feenberg 2000), and sensory reductionism (Tordsson 1999), which has led the analyses of technology from a classical phenomenological approach to remain fairly abstract, while placing a stronger emphasis on the ways in which technology alienates humans from their living environment. For example, Dreyfus and Spinoza (2003: 341) write that, despite Heidegger’s attention for human-technology relations, he considered technology’s greatest danger to be ‘a new totalizing style of practices that would restrict our openness to people and things by driving out all other styles of practice that enable us to be receptive to reality’. They caution that when technology becomes our primary way of experiencing the world, other ways of experiencing or understanding may disappear (Dreyfus and Spinoza 2003). These apprehensions towards technological determination also illustrate the concern of outdoor educators that technology may pose a barrier between learners and the natural world (Beames 2017; Hills and Thomas 2020; Smith et al. 2016).

Whereas phenomenological inquiry has offered valuable contributions towards better understanding the uses and purposes of tools in human existence, phenomenological tool analysis, with its strong focus on how tools are used, has arguably reached a limit of usefulness to describe and more deeply understand the complex dimensions of technological mediation that regard digital and mobile technologies.

To illuminate my argument, I draw on the inquiry of Edwards et al. (2021), who adopted a phenomenological approach to explore participants’ experiences of the impact of smartphone usage on achieving the learning objectives in an outdoor orientation program (OOP). The reasoning for conducting the study was, as they write, that it ‘is currently unclear how the presence of smartphones on OOPs may influence the experience on these trips’ (330). In line with a traditional phenomenological perspective, their analysis of the findings concentrated precisely on how the participants *experienced* the use of smartphones, rather than interpreting and explaining *how* smartphones truly did mediate these experiences. The findings of Edwards et al. (2021) strongly focus on the experience of smartphones as distractive, yet they fail to explain exactly *how* the use of these devices resulted in such distraction. That is beyond the superficial illustrations that participants were ‘scrolling on social media’ and experienced ‘stress’ and ‘anxiety’ because they were concerned about their phones (336).

Another finding regarded the influence of smartphones on the development of social connections. The conclusion drawn from this by Edwards et al. (2021) was that it remains ‘unclear if smartphones enhanced social connectedness or simply changed the manner in which these connections were fostered’ (338). I will return the examples of this inquiry later in this paper.

In short, the inability of phenomenology to adequately interpret contemporary relations and interactions between people, technology, and the environment may be problematic for educators and practitioners who deal with the same issues in practice. I argue that the increasing usage of digital and mobile technologies in outdoor education calls for more expanded theoretical underpinnings that allow the furnishing of more precise interpretations of mediated interactions between human, mobile technologies, and nature.

## Postphenomenological Mediation Theory

Wattchow (2001) stresses that pedagogical questions regarding the intentionality and mediation of any type of technology encountered in pedagogical practices should be raised and addressed. In the following section, I explore a postphenomenological perspective on technological mediation.

The American philosopher Don Ihde conceived postphenomenology as a philosophy of technology which addresses how humans and technologies are increasingly connected in today’s world (Ihde 1995, 2011). In the postphenomenological philosophy, Ihde builds on some of the core principles of phenomenology, but also breaks with it in significant ways. First and foremost, postphenomenology distances itself from the romantic tone present in classical phenomenology (Ihde 1995). Classical phenomenological writers have stated that phenomenology offers a rich alternative to the scientific approaches of analyzing phenomena from a distance and presenting a reduced reality and that phenomenology brings us ‘back to the things themselves’ (Merleau-Ponty 1962). Postphenomenology refutes this claim that phenomenology provides access to an original reality that is richer in meaning than the world presented by science and technology. Rosenberger and Verbeek clarify this as follows:

This means that postphenomenological claims are never about the absolute foundations of reality or knowledge, and never about the ‘essence’ of an object of study. Instead, postphenomenological claims are posed from an embodied and situated perspective, refer to practical problems, and are empirically oriented. (Rosenberger and Verbeek 2015: 1)

Put differently, postphenomenology resists the transcendental perspectives of the major phenomenological writers of the nineteenth and twentieth century who sought to understand the emerging meanings of experiences and phenomena as they appear, rather than the phenomena in themselves (Moustakas 1994). In contrast, postphenomenology positions itself as a ‘non-transcendental’, ‘non-foundational’, and ‘interrelational’ phenomenology (Ihde 1995: 1). This exemplifies the empirical turn and hints at the pragmatist perspective that Ihde incorporated into postphenomenological philosophy.

Ihde does, however, maintain the traditional phenomenological focus on intentionality and the roles of perception and embodiment in our experience of the world (Ihde 1995: 3). Intentionality plays a central role in conceptualizing human-world relations in both phenomenology and postphenomenology. Verbeek (2015) explains that, from a phenomenological perspective, humans are always directed towards something: we do not merely ‘see’, but we see something; we do not simply ‘feel’ but always feel something. Ihde takes this a step further by looking at the extension of perceptual and bodily intentionality through engagement with technological artifacts (Ihde 2011). This extension of the senses acknowledges that technological devices have become appropriated by our corporeal habits and that they allow people to undertake experiences beyond the unmediated experiences that are within the limited reach of physical movement and communication. For instance, *Season Traveller* is a virtual reality (VR) application in which users can move through four seasonal landscapes. Aside from the standard visual VR display, the application also incorporates sensory features, such as temperature, wind, and odor (Kerruish 2019). This illustrates just how advanced modern technologies are, but it also signifies the cultural significance of the dimensions of sensory perception.

In other words, technological tools do not merely function as instruments, but as mediators of individuals’ perception (Röhl 2018) and action (Verbeek 2006). The concept of *technological mediation*, inspired by Vygotsky’s (1986) theory of tool mediation, is the central epistemological position of postphenomenology. It aims to gain insight in the ways in which technology actively co-shapes the relation between people and the world through various mediating effects. De Boer et al. (2018) explain that this understanding of technological mediation emphasizes ‘the primacy of the relatedness between people, technologies and the world’, and that ‘postphenomenologists endorse the “co-constitution” of people and their material environment’ (300). They further clarify that ‘“co-constitution” means that, rather than existing independently, the relevant features of a person, a technological medium and the world appear as a result of their mutual relatedness’ (De Boer et al. 2018: 300). According to Ritter (2021b), this is the value of postphenomenology: it focuses on technological mediation and the ‘diverse effects of particular technologies instead of speculating on the essence of technology and its general impact’ (1). Before I delve further into the dimensions of technological mediation, I will outline and address some principal critiques on postphenomenology.

Postphenomenology has faced a myriad of critiques since its relatively recent emergence. For instance, Sparrow (2014: xiii) critiques the lack of a coherent center and states that it fails to ‘adequately clarify its methods, scope and metaphysical commitments’. Other scholars, such as Rosenberger and Verbeek (2015) and Aagaard (2017), acknowledge that postphenomenology is by no means perfect, but are working towards developing stronger empirical methods. Rosenberger and Verbeek (2015) attempt to advance postphenomenological research methods in their book *Postphenomenological Investigations: Essays on Human–Technology Relations*. Similarly, Aagaard (2017) offers empirical postphenomenological fieldwork as a means to explore the typical use of a given technology in-depth and to critically compare multiple types of technology.

Ritter (2021b) offers a critique on postphenomenology that is more relevant to this paper. Similar to postphenomenologists’ critiques of phenomenology, Ritter argues that postphenomenology’s ontological approach to technology and technological mediation remains ‘too abstract and speculative’ (591). He finds fault in the way in which postphenomenology overtly focuses on revealing the pragmatic relations between humans and technologies, therein reducing technologies to their practical functioning, rather than aiming to understand how the inclusion of technologies influences people’s intentionality. Besides, Ritter underscores that this entails paying attention to the instrumental values and functions that are visible, but, equally important, to the ‘pragmatically “invisible” parts of them’ (Ritter 2021b: 591). In other words, it is important to also understand the unintended side effects of using technologies.

Likewise, Kiran (2012) emphasizes that, while technologies can afford various actions, the use of such technologies can, in turn, have unexpected side effects. In postphenomenological terminology, this points at the multistability of a device: Technologies can have multiple functions depending on how people choose to use them (Ritter 2021b). Subsequently, Ritter (2021b) emphasizes that technologies can not solely mediate actions by being used in different ways, but also as side effects to how they are used. Ritter concludes that ‘postphenomenology should not focus on the pragmatically mediated, and thus multistable technicity of things’, but instead on how they mediate the relation between the subject and the object (597)—which for the purposes of this inquiry is the learner and the social and/or natural environment. Mediation theory can thus be useful in terms of explaining how technologies co-shape actions and experiences and mediate human-world experiences—whether intended or unintended.

In short, the contribution of the postphenomenological perspective may, at least, be considered as complementary to traditional phenomenological principles, based on its focus on practical problems (Rosenberger and Verbeek 2015), and potential to describe and anticipate various dimensions of technological mediation (Kiran 2015; Ritter 2021b). The next section will elaborate on this, by drawing on the use of mobile technology in the context of outdoor education.

## Kiran’s Framework for Technological Mediation

Aagaard (2017) argues that technological mediation is a vital, yet often neglected, aspect of pedagogical practices and calls for research that explores the typical use of a certain type of technology in depth. To gain a deeper understanding of the mediating impact of mobile technologies, I draw on Kiran’s (2015) framework of the four dimensions of technological mediation: ontological, epistemological, practical, and ethical. Each of these dimensions consists of a shaping movement and a downplaying one, which will be clarified in the next section. Kiran (2015) refers to this as the ‘two-sidedness’ of technological mediation. This two-sidedness does not imply a question of whether it is ‘good’ or ‘bad’ or if we can choose whether it should enter our lives or not, but *how* we can co-shape our lives in relationship with this technology. In other words, the term does not denote a binary; it rather refers to how a phenomenon can carry multiple characteristics at the same time. The two-sidedness of technological mediation can then be understood as a multiplicity of possible mediating movements. The term ‘multiplicity’ is inspired by Barad’s (2007) perspective on a multiplicity of possibilities, as she explains that ‘statements and subjects emerge from a field of possibilities. This field of possibilities is not static or singular but rather is a dynamic and contingent multiplicity’. (147) Exposing the two-sidedness by means of a multiplicity of mediating impacts contributes to an important broadening of the basis on which we can more deeply understand the impacts of technologies (Kiran 2015: 124–25), both on a collective level and on an individual one, and move away from the nature-technology binary.

### Ontological Dimension

First, at the ontological level, technologies co-shape the places in which we are situated and our experiences of these places. This is referred to as *revealing-concealing*. The relationship between mobile technologies and place is of particular importance because mobility is key to such technologies; they are always situated in a place. This occurs most obviously through the location and mapping applications that underpin mobile devices (Wilken and Goggin 2012: 4). De Souza e Silva and Sutko (2011) suggests that location-aware mobile media and the increasing use of navigational and/or place-specific applications effectively interweave the physical and the digital—dissembling the dualism, as both come together in ‘the immanence of the real’ (34).

In outdoor education, the use of location-based games, such as geocaching, has become increasingly popular (Farman 2009; Schaal and Lude 2015). Farman (2009: 3) argues that the users of location-based services navigate the landscape in a ‘simultaneous process of sensorial movement through streets and buildings and an embodied connection to how those places are augmented by digital information on mobile devices’. This view is further explained by de Souza e Silva:

Because many mobile devices are constantly connected to the Internet . . . users do not perceive physical and digital spaces as separate entities and do not have the feeling of ‘entering’ the Internet, or being immersed in digital spaces, as was generally the case when one needed to sit down in front of a computer screen and dial a connection. (De Souza e Silva and Sutko 2006: 263)

This mergence, now described as ‘postdigital’ (Jandrić et al. 2018), increasingly constitutes the outdoor education context.

### Epistemological Dimension

The second dimension involves *magnification reduction,* which lies at the epistemological level (Kiran 2015: 128). This dimension is crucial to understanding the postphenomenological interactions of mobile technology and our accounting for fluctuating levels of attentiveness to people and the physical environment (Wilken and Goggin 2012: 14). At this level, mobile technology affords certain perceptual capabilities while at the same time reduces other aspects of our experiential presence (Ihde 1979: 9).

Augmented reality (AR) applications can modify and even redefine learning activities (Romrell et al. 2014). For example, the ViewRanger Skyline application uses GPS services to generate and present hiking and biking trails. The application can also use the device’s camera to identify peaks using augmented reality, and it has a three-dimensional flyover feature through which the use can see and explore trails (Augmentra Ltd 2006). Such an AR representation of the landscape allows user to enter a different experience of the environment and perceive contextual features which otherwise remain hidden, unless they are physically within reach. Thus, mobile AR applications offer new ways of accessing and representing contextual information, and therefore knowledge, in the outdoors, which one would not be able to explore otherwise (Frajberg et al. 2017). In this sense, the mobile device enacts particular ways of seeing and thus knowing. The field of outdoor education could arguably benefit from encouraging learning in and through more broadly conceived domains of experience.

### Practical Dimension

In the third dimension, mobile technology co-shapes actions and behavior on a practical level through its *enabling-constraining* abilities (Kiran 2015: 131). While mobile technologies can increase our ability to engage with the environment in a particular way, this is accompanied by a reduced ability to engage with it in other ways.

To return to the example of location-aware functions, navigation applications, like ViewRanger, AllTrails, and Gaia GPS, enable us to navigate and explore features in the landscape in ways that were previously impossible. At the same time, the easy access to such applications may constrain the development of basic skills, like navigating with map and compass (Loynes 2020). This is an important consideration, as the development of cartographic literacy skills is still a common objective in outdoor education (Hergan and Umek 2017; Loynes 2020).

Hergan and Umek (2017) conducted a case study in which they compared navigation with paper maps and mobile navigators. The findings show that 87.7% of the students completed the route successfully with the help of a mobile navigator, while only 1.6% completed the route independently with the paper map. Most errors were made in choosing the right direction at crossroads (96). The egocentric spatial positioning was mentioned as one of the core advantages using the mobile navigator, because it automatically adjusts the environment to the participants’ movements. Nevertheless, some participants expressed that the features that change the display (e.g., from egocentric to allocentric or from two-dimensional to three-dimensional spatial perception) were distracting (Hergan and Umek 2017: 103). It is important to note that the case study was done with a group of 10-year-old students. Subsequently, it can be interpreted that successful experiences with technology-enhanced learning depends on the skill level of target groups and whether they are able to utilize and benefit from the different navigational features. Conversely, some participants stated that using the paper map encouraged them to be more observing and attentive to the environment. The findings also showed that multiple participants preferred using the paper map because they experienced a stronger sense of accomplishment when they made correct decisions (Hergan and Umek 2017: 104). This sense of mastery and skill development is an important feature of outdoor education (Beames et al. 2019; Beames and Brown 2016). The following quote by Casey (2012) illustrates the ambivalence of mobile navigators in terms of convenience, mastery, and risk:

But if I go into wilderness with a GPS backup to ensure my rescue if anything life-threatening should occur, have I not surrendered one of the most valuable aspects of going to the wilderness in the first place? Am I subtly less present, less alert to situations of danger and thus less attuned to the intelligence those situations would solicit? Will I become less able to deal with risks elsewhere in life? (Casey 2012: 177)

Indeed, even if one does not use the mobile device, its availability may still unintendedly co-shape our actions (Rosenberger and Verbeek 2015: 18–19). Hence, the anticipation of having a mobile device ready-at-hand can prime individuals to take more risks in the outdoors due to a technology-driven false safety (Beames 2017; Cuthbertson et al. 2004).

While it certainly is important to take such risks into account, it is not the technological tool itself that creates precarious situations. Wyatt (2008) argues that it is important not to fall in the trap of absolving individuals of their actions by putting the ‘blame’ on technology. Instead one should acknowledge the reciprocal human-technology relationship and the ability of themselves and other individuals to make well-considered choices (Matthews 2021). In this regard, educators may consider discussing the circumstances under which participants can use technologies, as well as the challenges connected to such usage.

### Ethical Dimension

Finally, Kiran’s (2015) ethical dimension points towards the *involving-alienating* effect of technologies. At this level, technologies do not change just what we perceive objectively, but also ‘*how* we are—perceptively—in the world’ (Ritter 2021a: 14). Mobile technologies are assessed in terms of the opportunities and hindrances they pose (Kiran 2015: 136), for example, to influence learners’ mobility in conceptual space. That is how mobile technologies may mediate learners’ shifting awareness and sense of presence in the here and now.

The term ‘mobility’ has long been understood as an individual body’s movement between locations and within a specific geographical space. Yet, in today’s postdigital world, it becomes more and more difficult to define space in terms of the dichotomized classifications of near/far, here/there, and presence/absence (Thompson 2016). The argument that digital and mobile technologies have fragmented the physical spatial relation into disconnected spatial and temporal connections (Richardson 2007) raises concerns among outdoor educators who seek to offer an ‘offline’ experience. Urry (2007: 7), however, does not merely understand mobility in terms of geographical movement, but instead defines the term ‘mobile’ as ‘something that moves or is *capable* of movement’. The core feature of mobility is thus movement, which can take shape in a variety of ways, and may involve people, information, or objects (i.e., mobile devices). From this perspective, it can be argued that mobile technologies simply expand opportunities for conceptual mobility and shifting attention.

Nevertheless, extant literature expresses clear concerns regarding the alienating impact of digital and mobile technologies. Returning to the example of Hergan and Umek’s (2017) study, the participants stated that navigation with a paper map required them to take more time to identify and observe features in the landscape, and thus, they were more attentive to the environment. Conversely, their experience with the mobile navigator was more efficient, but also drew more attention to the device than it did to the environment in which the activity took place. These findings are similar to the findings of Edwards et al. (2021), which I drew on to illustrate the inadequacy of phenomenology to interpret the mediating impact of mobile technologies. I will now expand my discussion of their findings in light of the involving-alienating impact.

The participants in the study of Edwards et al. (2021) described that the use of smartphones was experienced as distractive, and some participants experienced stress due to the feeling of having to keep in contact with people outside the trip. The allurement of smartphones to ‘go online’ or communicate with an absent person is an example of Margaret Morse’s (1998: 100) ‘ontology of everyday distraction’. This concept refers to a decrease of attentiveness to the here-and-now and a partial absent-minded-ness or ‘spacing out’. It is also tied to the concern from the traditional phenomenological perspective that technologies may become our principal or preferred way of knowing the world, as discussed earlier. Correspondingly, it is one of the concerns of outdoor educators that this shifting attention of participants between here-and-now and virtuality (elsewhere), defined by Morse as ‘nonspace’ (Morse 1998: 107), can alienate participants from the social and natural environment in a pedagogical setting. Similarly, Stiegler (1998, 2009) has argued that modern technologies engender a loss of individuals’ situated, embodied experience of space, place, and time, and that this reduced situatedness leads to a disorientation of contemporary experience. The concerns addressed here point precisely at the side effects that Ritter (2021b) calls attention to when it comes to a further development and application of postphenomenological mediation theory. However, rather than arguing that mobile technologies engender a loss of individuals’ embodied experiences, as Stiegler (1998, 2009) suggests, I emphasize that mobile technologies add new layers of complexity to learning situations through multiple of co-shaping movements. In some cases, they may cause distraction; in others, they may enrich learning experiences. Regardless, learners remain embodied and situated.

Various studies certainly show that the use of mobile devices can also have an involving effect on people’s experience of the social and natural environment. For example, mobile technology can strengthen participants’ involvement, in this case with the natural environment, through providing immediate access to information about, for instance, local flora and fauna (Hougham et al. 2018; van Kraalingen 2021; Zimmerman et al. 2019). Geochaching, MyNature Animal Tracks, iBird, iNaturalist, and Picture Insect are but some examples of educative applications that can elicit people’s interest in learning about the natural environment.

In short, from the postphenomenological perspective presented herein, mobile technologies should not be simply labeled as ‘good’ or ‘bad’. Instead, Ihde (1995: 33) writes that ‘[technologies] are transformational in that they change the quality, field and possibility range of human experience, thus they are non-neutral’. Then, we should not merely examine the changes mobile technologies cause in terms of a loss of connection to place, as I have extensively exemplified, but rather the ways in which they mediate learners’ experiences.

Although Kiran’s (2015) four-fold framework has the potential to help us more deeply understand the mediating impact of mobile devices, it does not assist in understanding technological mediation within the wider context of the changing nature of teaching and learning. Furthermore, it does not specifically explicate the technological mediation of human–human interactions, which is considered an essential part of the social dimension of learning (Dewey 1938; Slavin 1996). Hence, one can find limits in the direct translation of the postphenomenological mediation theory to an educational context.

## Technological Mediation and Outdoor Learning

In the following, I introduce a novel conceptual framework for technological mediation and outdoor learning (TMOL), inspired by the core features of phenomenological mediation theory, as outlined by Kiran (2015). I will illustrate the TMOL framework using examples from the outdoor education context.

There are two principal benefits of this framework. First, it considers the dimensions of postphenomenological mediation in a pedagogical context by incorporating a socio-constructivist perspective on learning. From this perspective, knowledge and learning experiences are constructed through interactions in the (outdoor) classroom (Adams 2006; Ceratto-Pargman et al. 2018; Watson 2001). The importance of social processes, interaction, and collaboration is underscored in educational literature (Dewey 1938; Slavin 1996), outdoor educational research (Beames and Atencio 2008; Becker et al. 2017), and social-cultural and constructivist perspectives on mlearning (Ceratto-Pargman et al. 2018; Kearney et al. 2012). Thus, the framework does not merely focus on the technological mediation of human-world relations, but also includes the social dimension. The underlying assumption is that learning cannot be understood separately from the social and natural environments in which it is formed (Adams 2006). Moreover, Lonchamp (2012) argues that the use of socio-cultural tools (i.e., mobile technologies) in education is based on the supposition that educators intentionally construct instrumented learning situations for specific pedagogical purposes. In light of the above, the TMOL framework foregrounds interactions and pedagogy, as opposed to placing focus on technological instrumentality and determination.

Second, the framework incorporates the two-sidedness of technological mediation to offer a nuanced perspective on the complexities of the mediating impact of mobile technologies. Hence, the TMOL framework acknowledges the downplaying effect and what may get ‘lost’ by using technologies (i.e., unintended side effects). In this regard, there is space to consider the deliberate avoidance of mobile technologies and preserving some of the traditional values and practices of outdoor education.

**Fig. 1:**
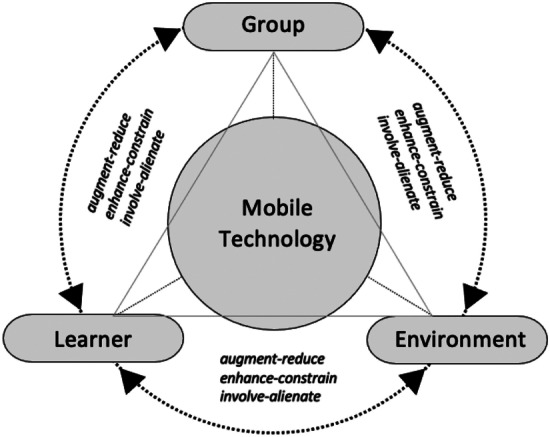
Technological mediation and outdoor learning framework (TMOL)

As shown in Fig. 1, mobile technology is placed at the core of the framework, as mediator of the relations between the individual learner, the group (social environment), and nature (natural environment). Each of these interactions involves three potential mediating effects: augment-reduce, enhance-constrain, and involve-alienate.

It should be noted that the framework does not include the ontological dimension included in Kiran’s (2015) framework. While this dimension has contextual significance in terms of understanding the role of connectedness in outdoor education, it is not the aim of this paper to explore what *is* (i.e., how mobile technologies co-shape the outdoor classroom ontologically). Next, I will discuss the TMOL framework using examples from the case of digital field diaries.

The use of digital field diaries as a visual method is commonly adopted in both research and education (Cooley et al. 2014; Volpe 2018). Similar to other visual methods, such as photo-elicitation, critical dialogue about a certain theme supported by visual information is often ‘used to describe the process of using photographs or pictures to tell a story’ (Volpe 2018: 363). To further illustrate the use of digital field diaries in outdoor education, I will sketch a hypothetical case with the learning objective to create a collaborative video diary about foraging edible plants. Foraging is considered a life skill that can also contribute to enhance people’s understanding of the local, natural environment (Webster 2019).

First, augment-reduce points at the extension or reduction of learners’ sensory perception of the environment. For example, the camera can function as an embodied extension of the learner’s perceptual ability (De Klerk 2020). Learners can zoom in to augment particular features of edible plants, such as the gills of mushrooms, which are important for species identification. Conversely, the orientation of learners’ intentionality through the lens of the device can reduce their visual perception of the wider surroundings, for instance, when leaners’ immediate perceptual attention (intentionality) is directed to the device or what is seen through it, or when learners focus on recording each other. This illustrates Kiran’s (2015) and Susser’s (2017) claims that the immediate focus on experiencing a phenomenon through a device reduces one’s acquisition of sensory information of what can be heard, seen, smelled, tasted, and felt in the wider surroundings.

Second, mobile technologies can enhance or constrain practical learning activities through the affordances of the devices. For example, making digital field journals can enhance learners’ knowledge and understanding of edible plants through capturing images of certain plants and then researching their qualities online. Through this documentation, experiences can be shared or recalled later in time (Ardoin et al. 2014), therefore extending the spatial and temporal elements of what otherwise may have been bounded learning experiences.

With regard to the learner’s interaction with the group, creating video diaries can enhance collaboration skills, social interaction between learners, and open opportunities for knowledge sharing. Kearney et al. (2012) write that, from a social-cultural and social-constructivist perspective on mlearning, learning is developed through social interactions as well as mediated through the use of technology. They argue that mobile technologies are conductive to collaborative and rich peer interactions. In accordance to this, the studies of Cooley et al. (2014) and Fuller and France (2016) show that video diaries enhanced teamwork due to the need for discussion and negotiation within the group. Additionally, learners can acquire new skills related to the use of mobile devices (Van Laar et al. 2017).

The final dimension regards the involving-alienating impact in relation to the group and the natural environment. At this level, it is important to consider the direction of learners’ intentionality (i.e., towards the device or towards the outdoor classroom) and their sense of presence. Mobile technologies can be used interactively, for example, to engage with the group through collaborative activities or knowledge sharing. Findings from Cooley et al. (2014) and Fuller and France (2016) indicate that the task fostered a greater sense of group identity and cooperation. Hence, it can be interpreted that digital field journals can have an involving impact between learners. Oppositely, an unintended side effect may include learners using the mobile device to communicate with people outside the learning setting (Bolliger and Shepherd 2017), which may cause an alienating effect and result in learners being physically together, but (virtually) connected elsewhere.

In regard to the learner’s relation to the environment, learning about local flora through video diaries can foster a student’s engagement with the natural world (Cooley et al. 2014). On the other hand, recording the learning experience may lead to learners being fully immersed in the device (Hills and Thomas 2021). When the learner’s focus shifts to what is seen through the camera, their awareness of self and physical presence in place, including the people in it, fades to the background. This effect is explained by Rosenberger and Verbeek’s (2015) notion of field of awareness. Rosenberger and Verbeek argue, the user enters ‘another world through the device’ (Rosenberger and Verbeek 2015: 23–24), referred to earlier as ‘spacing out’, which may lead them to being disconnected from the present experience.

**Fig. 2:**
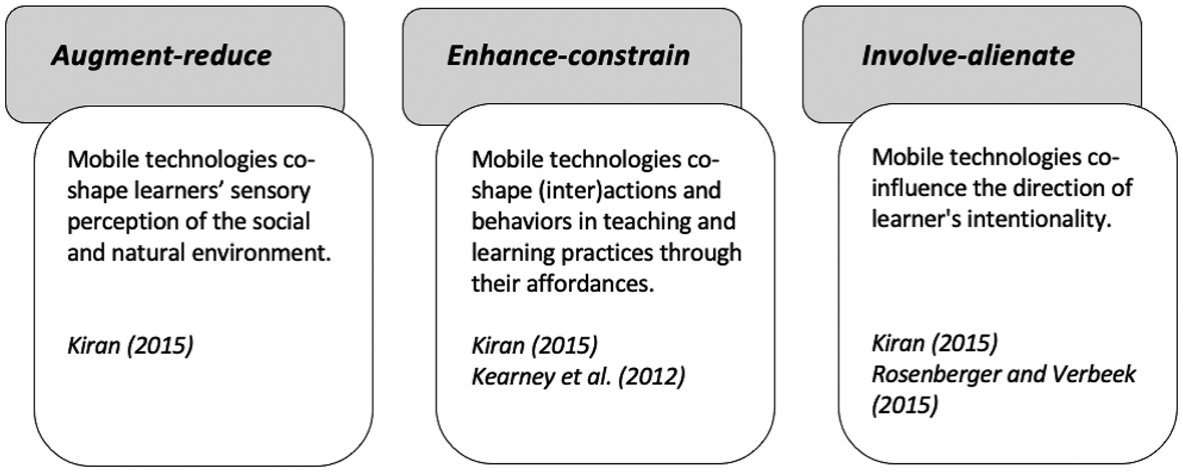
Synthesis of the dimensions of technological mediation in the TMOL framework

The two-sidedness of technological mediation, synthesized in Fig. 2, could be interpreted as being equivalent to the nature-technology or ‘pro-tech’ and ‘con-tech’ binaries. However, I emphasize that the different dimensions of mediation merely aim to give insight into the multiplicity of mediating movements that may be at play. Accordingly, some shaping movements and other downplaying movements can happen simultaneously. This aligns with broader postdigital perspectives that recognize the simultaneity and ‘entanglements between the online and offline, analogue and digital, and biological and informational’ (Traxler et al. 2022: 508). Thus, the framework aims to contribute to a more precise method for understanding which mediating movements are occurring in certain outdoor learning activities. This can assist educators in anticipating and understanding how mobile technologies might influence the direction of educator’s or learners’ intentionality.

The TMOL framework should be considered in relation to the learning objectives of specific activities. Through this paper’s theoretical inquiry, I suggest three guiding questions for educators:

- To what extent does the use of mobile technologies hinder the learning objectives, in terms of reducing, constraining, or alienating effects?
- To what extent does the use of mobile technologies complement the learning objectives, in terms of augmenting, enhancing, or involving effects?
- What is the added value of the use of mobile technologies?

A final remark worth mentioning is that the mediating impact of mobile technologies in pedagogical contexts is, at least in part, dependent on the competence of educators and the degree to which they carefully employ mobile devices to enhance student learning in very specific ways (Ceratto-Pargman et al. 2018; Hills and Thomas 2021; Lonchamp 2012). However, as it is neither the aim of this paper to address educators’ digital competence nor how educators *design* learning activities supported by mobile technologies, I will leave this open for future research.

## Conclusion

The rapid advancement of digital and mobile technologies does not only influence current practices in outdoor education, but also challenges the very nature of outdoor education itself. The employment of mobile technologies in education without equipping educators and participants with the ability to understand and respond to the mediating impacts of such devices would be short-sighted. As scholars and educators in the field of outdoor education, we can come one step closer to overcoming the rather unhelpful nature-technology binary by acknowledging that, in today’s postdigital context, mobile technologies play an active role in co-shaping learners’ experiences of the world, and not merely in terms of creating distance between learners and the natural world.

This paper set out to explore the potential of a postphenomenological perspective on mediation theory to help more deeply understand the mediating impact of mobile technologies on outdoor learning experiences. While the four-fold framework of technological mediation offered by Kiran (2015) can be considered a starting point for understanding the two-sidedness of the mediating impact, it does not consider technological mediation in pedagogical contexts. By drawing on postphenomenological mediation theory and basing itself on socio-constructivist perspectives on learning, this paper offers a novel framework for making more exacting sense of the complex, embodied human-technology-world relations in outdoor education.

The TMOL (technological mediation and outdoor learning) framework outlines three dimensions of technological mediation between the individual learner, the group, and the natural environment: augment-reduce, enable-constrain, and involve-alienate. Through considering the side effects of mediation, it can be interpreted that some traditions may be worth preserving based on the value of outdoor education as alternate setting in today’s postdigital reality. This framework is the result of a deep dive into extant theory and can be considered a helpful tool to gain insight into the multiplicity of mediating impacts that may be at play when mobile technologies are employed in outdoor education.

While technological mediation should be understood and analyzed within a situated context, it is suggested that the field of outdoor education could benefit from reflecting on the three types of mediation when adopting mobile technologies into outdoor teaching and learning activities. Further empirical research is needed to gain insights into learners’ and educators’ experiences of the technological mediation of mobile devices, as well as into the wider pedagogical effects within specific outdoor learning contexts. Finally, future inquiries may also focus on the technological mediation of emerging AR and VR technologies and the potential consequences of these developments for outdoor teaching and learning.
